# The effectiveness of an online educational program on nurses’ electrocardiogram interpretation skills

**DOI:** 10.1186/s12912-025-02997-y

**Published:** 2025-03-27

**Authors:** Gülşen Kılıç, İnci Mercan Annak, Hafize Savaş, Belma Ay Kılıçaslan, Merve Erdoğan Erener, Ahmet Arslan

**Affiliations:** 1 Department of Nursing, Faculty of Health Sciences, Baskent University, Ankara, Turkey; 2 Faculty of Nursing, Gazi University, Ankara, Turkey; 3 Department of Nursing, Faculty of Health Sciences, Lokman Hekim University, Ankara, Turkey; 4 Vocational School of Health Services, Lokman Hekim University, Ankara, Turkey; 5 Internal Intensive Care Unit, Ankara University Ibni Sina Hospital, Ankara, Turkey; 6 Department of Cardiology, Gülhane Training and Research Hospital, Ankara, Turkey; 7 Baskent University Bağlıca Kampüsü, Fatih Sultan Mahallesi, Eskişehir Yolu 18.km, Ankara, Turkey

**Keywords:** Electrocardiogram interpretation, Education, Electrocardiogram, Nursing

## Abstract

**Purpose:**

This study aimed to evaluate the effectiveness of an online educational program designed to improve nurses’ skills in interpreting electrocardiograms (ECGs).

**Methods:**

This study employed a single-group pretest‒posttest quasiexperimental design. The population consisted of 282 nurses who voluntarily participated in the online ECG education program. The five-hour online ECG education program covered six main topics: normal sinus rhythm and derivations, arrhythmias of sinus origin, arrhythmias of atrial origin, arrhythmias of ventricular origin, blocks, pacemakers, acute coronary syndrome, and myocardial infarction.

**Results:**

Participants had significantly higher mean posttest scores on the Electrocardiography Knowledge Test (ECG-KT) than did their pretest scores. Specifically, they achieved significantly higher posttest scores in the “normal sinus rhythm and derivations,” “atrial arrhythmias,” and “acute coronary syndrome” subcategories than did their pretest scores. The participants with intensive care certificates demonstrated significantly higher mean pretest and posttest ECG-KT scores than did those without such certificates. Additionally, participants with master’s or Ph.D. degrees had significantly higher mean pretest and posttest ECG-KT scores than those with bachelor’s degrees did.

**Conclusion:**

The results indicate that the ECG education program enhances nurses’ knowledge and improves their ability to interpret ECGs quickly and accurately. Therefore, universities and hospitals are recommended to offer online ECG education programs to support nurses in developing these essential skills.

**Supplementary Information:**

The online version contains supplementary material available at 10.1186/s12912-025-02997-y.

## Introduction

Since 2000, ischemic heart disease (IHD) has been among the top ten causes of death globally [1]. More than four million people in Europe die each year from cardiovascular diseases. Approximately 1.8 million of those deaths are due to coronary heart disease (CHD), whereas 1.1 million are due to stroke [2]. In Türkiye, circulatory system diseases (36.8%), excluding noncommunicable diseases, are the leading cause of death. When deaths from circulatory system disorders are broken down into their underlying causes, ischemic heart disease accounts for 39.1% of these deaths, cerebrovascular diseases account for 22.2%, and other heart conditions account for 25.7% [3].

Electrocardiography (ECG) is the gold standard initial test for diagnosing myocardial infarction (MI) and ischemia [4]. It is widely used because it is a noninvasive, easily accessible, and cost-effective method that provides immediate results [5]. Ward nurses often perform or monitor ECGs to address acute situations or administer special medications requiring ECG monitoring. Intensive care unit (ICU) nurses monitor ECGs to assess their patients hemodynamically. They also perform extra ECGs to follow up with their patients in acute situations. Therefore, it is crucial to correctly interpret ECGs to diagnose and treat serious dysrhythmias that increase the risk of death, such as myocardial ischemia, fluid‒electrolyte imbalances, and long QT syndrome. It is also crucial to recognize any sudden changes in the ECG that may occur because of the patient’s condition to take immediate action [6, 7]. 

Nurses are the primary caregivers who manage treatment processes 24/7. They are responsible for performing and monitoring ECGs to follow up with their patients [6, 8, 9]. The Nursing Regulation published in the official gazette (No: 27515) on April 19, 2011, stipulates that nurses are responsible for monitoring ECGs and communicating with other healthcare professionals in emergencies [10]. 

Nurses play an active role in healthcare services. Therefore, they are expected to have the knowledge and skills to provide high-quality and effective care and plan and implement interventions to identify patients with altered health status [11]. However, Çelik et al. [6] reported that emergency room and ICU nurses knew little about ECG interpretation. Özoğul et al. reported that nurses who attended ECG training had a significantly higher mean posttest score (87.6) than pretest score did [11]. İbrahim et al. [12] 201 recruited ICU nurses and provided them with ECG training. They reported three critical results. First, nurses who knew little about arrhythmia learned more about it from training. Before the training, only half of the nurses could monitor patients, but this increased to 80% afterward. Similarly, the percentage of nurses who could perform and interpret ECGs rose from 60% before training to 90% after training.

There is limited literature that ECG training programs make nurses better at interpreting ECGs. The Turkish Society of Intensive Care Specialists Nursing Commission organized the fourth Basic Intensive Care Nursing E-Course in 2022. The feedback from more than half (57.8%) of the nurses who attended the e-course showed that nurses needed training in ECG.

This study investigated the effect of an online ECG education program (intervention) on nurses’ knowledge of ECG interpretation.

## Materials and methods

### Study design

This study adopted a single-group pretest-posttest quasi-experimental research design.

### Participants

**Fig. 1:**
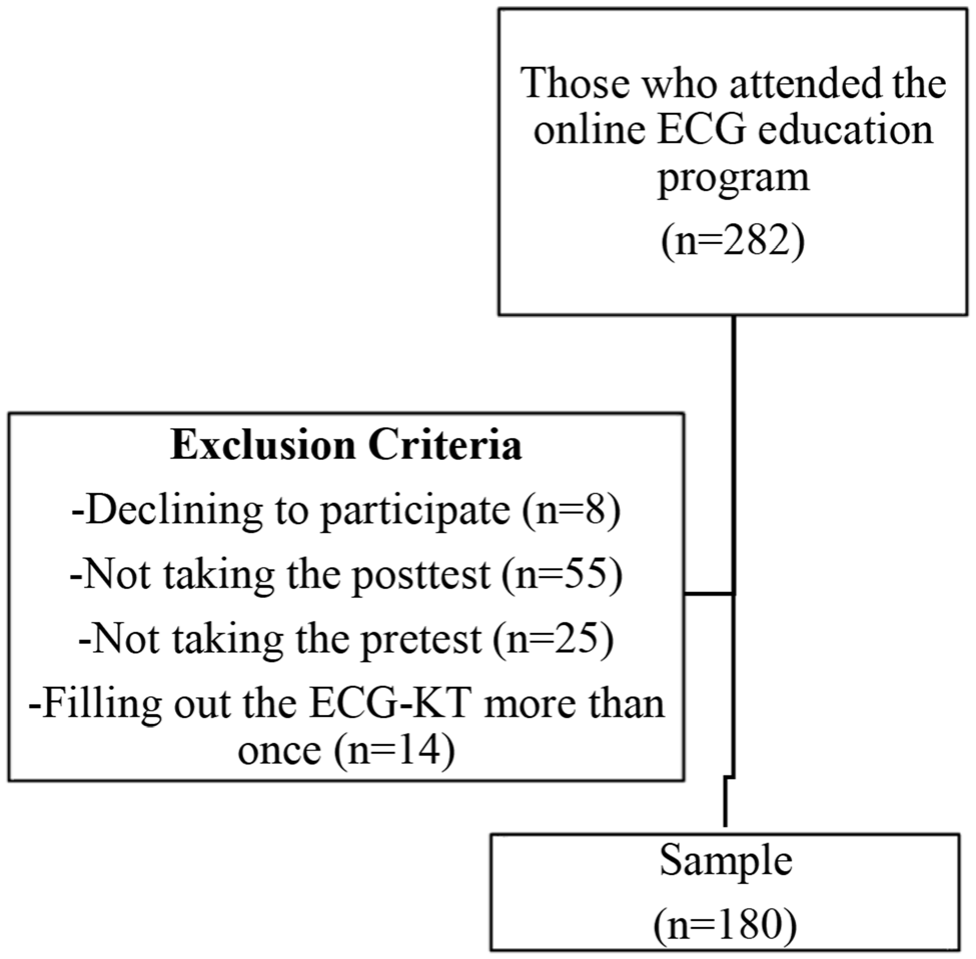
Population and sample

The study population consisted of 282 nurses who voluntarily attended the online ECG education program held by the Turkish Society of Intensive Care Specialists Nursing Commission. No sampling was performed because the study was designed to recruit as many nurses as possible. The sample consisted of 180 nurses who agreed to participate and met the inclusion criteria (Fig. 1).

### Inclusion criteria and exclusion criteria

The inclusion criteria were as follows: (i) attended the online ECG education program; (ii) volunteered; and (iii) filled out the ECG-KT as the pretest and posttest. The exclusion criteria were as follows: (i) completed the ECG-KT more than once and (ii) did not complete the pretest or posttest.

### Study instruments

#### Personal information questionnaire

The form consisted of seven items on age, gender, education, work experience, the unit of duty, ECG training, and intensive care certification.

#### ECG knowledge test

The Electrocardiogram Knowledge Test (ECG-KT) was developed by six researchers involved in the study. One of the researchers is actively working in the field of cardiology and participates in ECG analysis. Three of the researchers completed their theses in the field of cardiology, while the other two researchers work in intensive care within this area. The researchers developed the ECG Knowledge Test on the basis of their clinical experiences and literature reviews [6, 13–16]. After the questions were created, five experts reviewed the data collection forms for intelligibility and relevance. Based on the feedback from these experts, the final version of the test was prepared.

The ECG Knowledge Test consists of 20 items, each evaluated on a five-point scoring system (0–5). The total score ranges from 0 to 100. The items assess the respondent’s knowledge of ECG. The test consists of six subscales (normal sinus rhythm and derivations, atrial arrhythmias, ventricular arrhythmias, blocks, pacemakers, acute coronary syndrome, and myocardial infarction). The content of the items included general theoretical ECG knowledge and ECG interpretation. More detailed information about the ECG Knowledge Test can be found in the supplementary file attached to this manuscript.

#### Validity and reliability of the ECG knowledge test

The validity and reliability of the ECG Knowledge Test (ECG-KT) were evaluated through expert review and statistical analyses. To enhance content validity, five experts assessed the test items for intelligibility and relevance. Their feedback was incorporated into the final version of the test.

The reliability of the ECG-KT was evaluated using the test-retest method and item-total score correlation. Test-retest reliability was assessed by calculating the correlation between pretest and posttest scores. The Pearson correlation coefficient was *r* =.835 (*p* <.001), indicating a high level of reliability and consistency over time. Given this result, the ECG-KT demonstrates strong reproducibility and can be considered a reliable tool for assessing ECG knowledge.

#### Online ECG education program

The online ECG education program (intervention) was held online by four experts in the cardiovascular system. It consisted of four hours of theoretical information on ECGs and one hour of Q&A practice regarding ECG evaluation. The five-hour duration was chosen based on logistical feasibility and participant availability. Additionally, the duration was determined by reviewing the curriculum and course hours of undergraduate nursing education. The intervention covered the cardiac conduction system, normal rhythm and its characteristics, atrial arrhythmias, ventricular arrhythmias, blocks, pacemaker applications, acute coronary syndrome, myocardial infarction, and ECG interpretation. The pretest posttest questionnaire form was used to assess the effectiveness of the instructions provided in the ECG course.

### Data collection

The education was conducted online on October 11, 2022. Data were collected online via a personal information questionnaire and an ECG knowledge test (ECG-KT). The data were collected via a personal information questionnaire and an ECG knowledge test (ECG-KT).

Before the education, the researchers briefed all the participants about the research purpose and procedure. Then, they sent a link to the survey (Google Forms) as a text message online. All participants completed the ECG-KT (pretest). Then, they attended the intervention. After the intervention, they completed the ECG-KT (posttest), which was sent as a text message online. Each participant took 20–25 min to complete the ECG-KT (pretest and posttest). After the posttest, the experts and participants answered and discussed the ECG-KT items. Finally, the participants’ pretest and posttest scores were compared.

### Statistical analysis

The data were analyzed using the Statistical Package for Social Sciences (SPSS, v. 25.0) at a significance level of 0.05. Descriptive analyses included frequency, percentage, mean, and standard deviation. The Kolmogorov-Smirnov test was applied to assess normality, and the results confirmed that the data followed a normal distribution. Therefore, parametric tests were conducted, including independent-group t-tests, dependent-group (paired) t-tests, ANOVAs, and Tukey HSD post hoc tests. To further evaluate the results, effect sizes, including Cohen’s d, and post-hoc power analyses were also calculated.

## Results

Most participants were women (82.2%) between the ages of 20 and 29 years (71.1%) and had bachelor’s degrees (72.2%). More than half of the participants had 1–5 years of work experience (52.3%), with a minimum of one year and a maximum of thirty years of employment. More than half of the participants were ICU nurses (68.3%). Twenty-four participants were inpatient ward nurses (13.3%). Ten participants were emergency room nurses (5.6%). Four participants were surgical nurses (2.2%). Nineteen participants were students or academics (10.6%). Most participants had never previously received training in ECG (74.4%) (Table 1).

**Table 1:** Sociodemographic and occupational characteristics (*n* = 180)

Sociodemographic and Occupational Characteristics	*N*		%
**Gender**			
Woman	148		82.2
Man	32		17.8
**Age (year)**			
20–29	128		71.1
30–39	42		23.3
≥ 40	10		5.6
**Education (degree)**			
High school	15		8.3
Bachelor’s	130		72.2
Master’s or Ph.D.	35		19.5
**Work experience (year)**			
1–5	94		52.3
6–10	37		20.6
11–20	26		14.4
21–30	6		3.3
0 (student or academics)	17		9.4
**Working unit**			
Intensive care unit	123		68.3
Inpatient ward	24		13.3
Emergency room	10		5.6
Surgical	4		2.2
Not working as a nurse (student/academic)	19		10.6
**Having received training in ECG before**			
Yes	46		25.6
No	134		74.4
**Having an intensive care certificate**			
Yes	50		27.8
No	130		72.2

The participants had a significantly higher mean posttest ECG-KT score (66.2 ± 17.2) compared to the pretest score (46.5 ± 17.5) (*p* <.001). The posttest scores (M = 66.2, SD = 17.2) were significantly higher than the pretest scores (M = 46.5, SD = 17.5) (*p* <.001, Cohen’s d = 1.23). The effect size was calculated as Cohen’s d = 1.23, which is classified as a large effect size according to Cohen (1988) [17]. Additionally, a post-hoc power analysis was conducted, yielding a statistical power of (1 - β) = 99.9%.

They had significantly higher mean posttest ECG-KT “normal sinus rhythm and derivations” (*p* =.006), “arrhythmia of atrial origin” (*p* =.010), and “acute coronary syndrome” (*p* =.043) scores than did the pretest scores. They had the highest and lowest pretest scores for “arrhythmias of ventricular origin” (53.57 ± 21.41) and “blocks” (26.39 ± 1.17), respectively. They had the highest and lowest posttest scores on “pacemaker” (78.33 ± 10.21) and “blocks” (36.39 ± 1.17), respectively (Table 2). Age and sex did not significantly affect our participants’ pretest and posttest ECG-KT scores (*p* >.05) (Table 3).

**Table 2:** Pretest and posttest ECG-KT scores

Pretest and Posttest ECG-KT Scores	Pretest (X̄±SS)	Posttest (X̄±SS)	t/*p*	
**Subscales**				
Normal sinus rhythm and derivations	52.68 ± 24.02	75.09 ± 20.70	-4.638/0.006*	
Atrial arrhythmias	36.85 ± 11.65	60.55 ± 14.79	-9.849/0.010*	
Ventricular arrhythmias	53.57 ± 21.41	74.44 ± 11.64	-2.519/0.086	
Blocks	26.39 ± 1.17^a^	36.39 ± 1.17^a^	-	
Pacemaker	50.83 ± 0.38	78.33 ± 10.21	-3.957/0.158	
Acute coronary syndrome	44.81 ± 17.96	56.11 ± 22.03	-4.689/0.043*	
**Total**	46.5 ± 17.5	66.2 ± 17.2	-16.604/<0.001*	

X̄: Mean; SS: Standard deviation

^a^ Correlation could not be calculated because the standard error was close to zero

*Paired samples t test

**Table 3:** Pretest and posttest ECG-KT scores by sociodemographic characteristics

Sociodemographic Characteristics	Mean ECG-KT Scores (X̄±SD)	
	Pretest	Posttest
**Gender**		
Woman	45.89 ± 1.43	65.40 ± 1.38
Man	49.37 ± 3.18	70.00 ± 3.25
	t: 1.01 p:0.309*	t:1.37 p:0.172*
**Age (year)**		
20–29	45.09 ± 1.52	65.27 ± 1.56
30–39	50.11 ± 2.92	69.28 ± 2.53
40–49	49.5 ± 4.24	65.50 ± 4.62
	F:1.46 p:0.235*	F:0.866 p: 0.422*
**Education (degree)**		
High school^(1)^	47.33 ± 4.92	60.66 ± 4.85
Bachelor’s^(2)^	44.55 ± 1.57	65.00 ± 1.54
Master’s or Ph.D.^(3)^	53.19 ± 2.29	72.91 ± 2.28
	F: 3.53 p:.031** **Difference: 3 > 2**	F:3.95 p:.021** **3 > 2**
**Work experience (year)**		
1–5^(1)^	45.44 ± 1.80	66.01 ± 1.78
6–10^(2)^	51.89 ± 2.78	69.32 ± 2.60
11–20^(3)^	50.00 ± 3.72	67.30 ± 3.62
21–30^(4)^	47.50 ± 8.21	65.00 ± 4.28
0 (student)^(5)^	35.00 ± 3.45	59.41 ± 4.61
	F: 3.20 p: 0.014** **Difference: 2 > 5** **Difference: 3 > 5**	F: 1.002 p: 0.408**
**Working Unit**		
Intensive care^(1)^	49.04 ± 1.49	67.72 ± 1.48
Inpatient services^(2)^	39.37 ± 3.73	60.41 ± 3.69
Emergency services ^(3)^	51.00 ± 7.95	68.00 ± 6.79
Surgical services ^(4)^	50.00 ± 2.04	73.7 ± 4.73
Not working as a nurse^(5)^ (student/academics)	36.05 ± 3.18	61.31 ± 4.27
	F: 3.74 p: 0.006** **Difference: 1 > 5**	F: 1.53 p: 0.193**
**Having received training in ECG before**		
Yes	56.95 ± 2.34	72.06 ± 2.56
No	42.92 ± 1.43	64.21 ± 1.44
	t: 4.98 p < .001*	t: 2.71 p: 0.007*
**Having an intensive care certificate**		
Yes	54.04 ± 2.08	70.20 ± 2.13
No	43.44 ± 1.5	64.53 ± 1.56
	t: 3.75 p < .001*	t: 1.99 p: 0.048*

* Student’s t test

** one-way ANOVA

X̄: Mean; SS: Standard deviation

Education significantly affected our participants’ pretest and posttest ECG-KT scores (pretest *p* =.031; posttest *p* =.021). The participants with master’s degrees or Ph.D.s had significantly higher marks and posttest ECG-KT scores than did those with bachelor’s degrees (pretest *p* =.024; posttest *p* =.037). Work experience also significantly affected their pretest and posttest ECG-KT scores (*p* =.014). Nursing students had a significantly lower mean pretest ECG-KT score than did those with 6–10 years (*p* =.008) and 11–20 years of work experience (*p* =.043). The working unit also significantly affected their pretest and posttest ECG-KT scores (*p* =.006). Nursing students and academics had significantly lower mean pretest ECG-KT scores than ICU nurses did (*p* =.019). The participants who had previously received ECG training had significantly higher mean pretest and posttest ECG-KT scores than did those who had not (pretest *p* <.001; posttest *p* =.007). The participants with intensive care certificates had significantly higher mean pretest and posttest ECG-KT scores than did those without (pretest *p* <.001; posttest *p* =.048) (Table 3).

## Discussion

We provided nurses with the Fourth Basic Intensive Care Nursing E-Course between February 21 and 22, 2022. The course also addressed the topic of “common cardiac rhythm disorders in intensive care units.” At the end of the course, we asked all attendees what topic they would like to receive training in. Three of the five attendees said they would like to be trained in ECG (57.8%). Both the demands during the course and the feedback at the end of the course showed that nurses needed training in ECG.

Our participants had a significantly higher mean posttest ECG-KT score (66.2 ± 17.2) than the pretest score (46.5 ± 17.5) (*p* <.001), indicating that the online ECG education program (intervention) was effective. The effect size (Cohen’s d = 1.23) suggests that the intervention had a meaningful impact beyond mere statistical significance, highlighting its potential clinical relevance in enhancing nurses’ ECG interpretation abilities. Studies have demonstrated that structured education programs significantly improve nurses’ ECG interpretation skills and clinical confidence [6, 7, 9, 11, 16, 18, 19]. In line with this, our findings indicate that nurses with prior ECG training performed better, suggesting that ECG education should be periodically updated to maintain competency and reinforce skills.

Our participants had the highest pretest ECG-KT score for “ventricular arrhythmias” (53.57 ± 21.41), whereas they had the highest posttest ECG-KT score for “pacemakers” (78.33 ± 10.21). Coll-Badell et al. [19] reported that nurses are aware of ventricular tachycardia as well as flutter arrhythmias. In our study, a high rate of ventricular arrhythmias in the pretest was frequently encountered in intensive care units or services, and nurses recognized these rhythms.

Our participants had the lowest pretest and posttest ECG-KT scores on ‘blocks,’ consistent with findings from Ho et al. and Chen et al., who reported that nurses struggle with recognizing heart blocks and abnormal conduction patterns. This result suggests that additional targeted education focusing on identifying and differentiating heart blocks is necessary [20, 21]. This is probably because nurses care for fewer patients with heart blocks than those with other types of arrhythmias (normal sinus rhythm, atrial fibrillation, etc.). The lowest scores were on blocks in the ECG-KT. This suggests that the subject of blocks should receive greater emphasis in subsequent instruction.

Age and sex did not significantly affect our participants’ pretest and posttest ECG-KT scores. While Erişti et al. [7] reported similar results, Zhang et al. [18] and Coll-Badell et al. [19] did not perform any pretest or posttest comparisons in terms of age or sex. On the other hand, Çelik et al. [6] recruited nurses and provided them with a one-hour training program on basic ECGs. They reported that nurses between the ages of 23 and 27 answered significantly more questions correctly than other age groups did before and after the intervention. Rahimpour et al. [15] reported that female emergency room nurses were better at interpreting ECGs than their male counterparts were. On the other hand, Ho et al. [20] reported that male nurses had a significantly higher mean ECG interpretation score than their female counterparts did. These results indicate that more research is warranted to better understand the effects of age and gender on nurses’ ECG interpretation skills.

Our participants who had previously received ECG training had significantly higher mean pretest and posttest ECG-KT scores than those who had not (*p* =.007). Zhang et al. [18] investigated the effectiveness of an education program on nurses’ knowledge of ECG interpretation and reported similar results. Research, in general, shows that nurses who have received training in ECGs before they learn more about them than those who have not [18–20]. Our results suggest that nurses are willing to learn new things, as they know that maintaining good nursing skills requires continuous learning and review. Our results also revealed that participants with intensive care certificates had significantly higher mean pretest and posttest ECG-KT scores than did those without intensive care certificates. This result suggests that certification programs are effective ways to help nurses refresh their knowledge of ECG interpretation.

It is evident that education helps nurses acquire professional knowledge and develop professional skills [22]. Our participants with master’s or Ph.D. degrees had significantly higher mean pretest and posttest ECG-KT scores than those with bachelor’s degrees did (pretest *p* =.24; posttest *p* =.37), which is consistent with the literature [7, 11, 16]. For example, Özoğul et al. [11] reported that nurses with master’s degrees knew more about ECGs than did those with bachelor’s degrees. Erişti et al. [7] reported that nurses with master’s degrees were better at interpreting ECGs and recognizing deadly rhythms than others. These results suggest a positive correlation between education and ECG knowledge.

Practice makes knowledge more automatic and durable. Our results revealed that nursing students had a significantly lower mean pretest ECG-KT score than did those with 6–10 years (*p* =.008) and 11–20 years of work experience (*p* =.043). Tahboub et al. [23] also reported that nurses with less than one year of work experience knew less about ECGs than those with more than six years of work experience did, suggesting that nurses with more work experience are likely to have more ECG knowledge and skills. Although our results revealed that nursing students had higher mean posttest ECG-KT scores than pretest scores did, the difference was statistically insignificant (*p* >.05). This is probably because they are open to learning new things.

Nursing students and academics had significantly lower mean pretest ECG-KT scores than ICU nurses did (*p* =.019). Werner et al. [22] also reported that ICU nurses knew more about ECGs than other clinical nurses did. Although Erişti [7] reported that ICU nurses were better at interpreting ECGs and recognizing deadly rhythms than other nurses were, the difference was statistically insignificant. These results suggest that nurses working in critical units know more about ECG interpretation because they constantly monitor ECGs to follow up with their patients. Prior research has demonstrated that systematic training programs markedly enhance participants’ ECG interpretation abilities [9, 15, 19]. This study investigated the effectiveness of online training for nurses, and the results align with existing literature, indicating that the quality of the training process directly influences learning outcomes [16, 20]. These findings are consistent with previous studies on ECG education effectiveness, further supporting the importance of structured training programs in improving ECG interpretation skills [9, 15, 19, 20]. 

### Limitations of the study

This study has some limitations. The study does not include a comparison group, limiting its robustness. Future studies are recommended to include a control group to enhance the robustness of the findings. Additionally, the study mainly included ICU nurses and volunteers who already had a strong interest in ECG interpretation, which may have influenced the results. Furthermore, participants were not asked whether they had certificates other than intensive care certificates, which could be a limitation in assessing their prior knowledge. Another limitation is the short-term follow-up since the posttest was administered immediately after training, and knowledge retention over time was not evaluated. To address this issue, future studies should consider conducting follow-up assessments at 3 and 6 months to evaluate the long-term retention of ECG interpretation skills.

## Conclusion

This study shows that an online ECG training program can help nurses improve their ECG interpretation knowledge and skills. This is clinically significant, as nurses’ ability to interpret ECGs correctly and in a timely manner is crucial for patient safety. It allows for the early detection of life-threatening arrhythmias or ischemic signs, enabling faster intervention when necessary.

Our findings emphasize the importance of integrating structured ECG interpretation training into nursing education and ensuring regular professional development opportunities. Moreover, online ECG training appears to be a practical and effective learning tool, offering flexibility and accessibility, particularly for busy nurses who may struggle to attend in-person training.

Since studies suggest that ECG interpretation skills may decline over time, future research should focus on assessing the long-term retention of knowledge gained through online education and evaluating whether regular refresher courses help nurses maintain their competency in ECG interpretation.

## Electronic supplementary material

Below is the link to the electronic supplementary material.

Supplementary Material 1

## Data Availability

No datasets were generated or analysed during the current study.
