# Supplementary material for: The effectiveness of an online educational program on nurses’ electrocardiogram interpretation skills
Source: BMC Nurs. 2025 Mar 27;24:334. doi: 10.1186/s12912-025-02997-y (PMC11948722; doi:10.1186/s12912-025-02997-y)
Supplement: Supplementary file 1 — Supplementary Material 1 [file 12912_2025_2997_MOESM1_ESM.docx]

**ENHANCING ELECTROCARDIOGRAM INTERPRETATION SKILLS: PRE-TEST AND POST-TEST QUESTIONS FOR NURSES**

1. **Which of the following is the correct sequence of waves in an electrocardiogram with a normal rhythm?**

a) PRSTQ wave

b) QRSTP wave

**c) PQRST wave**

d) PSTQR wave

e) QSPRT wave

**2. Which of the following represents depolarization of the ventricles?**

a) P wave

b) Q wave

c) R wave

d) PR segment

**e) QRS complex**

**3. If the number of small squares between two QRSs in an electrocardiogram with a regular rhythm is 20, which of the following is the pulse value?**

a) 55 beats/min

b) 60 beats/min

**c) 75 beats/min**

d) 80 beats/min

e) 90 beats/min

**4. Which of the following is not an ECG feature in normal sinus rhythm?**

a) Heart rate 60–100 beats/min

b) There are clearly observable P waves.

c) Each P wave is followed by a QRS complex.

**d) PR interval at most 0.50** **sn**

e) PR interval is equal in all cycles

**
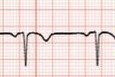
**

**5. In the picture, there is an image of a normal ECG. Which derivation below could this image belong to?**

a) V4

b) V5

**c) aVR**

d) aVF

e) DII


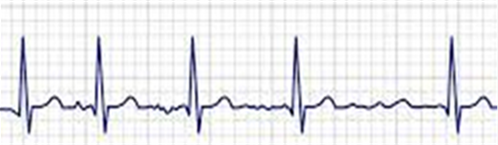


**6. Which of the statements regarding the arrhythmia whose ECG image is given in the figure above is incorrect?**

1. It is an irregular and rapid rhythm in which stimuli arise from multiple ectopic foci in the atrium.
2. No discernible P waves; irregular wavy waves are seen. PR interval cannot be measured.
3. Deficit pulse is seen
4. There is a constant thrombus in the atria and therefore a risk of embolism.
5. Intravenous amiodarone is primarily administered in treatment.

I. Occurs when the sinus node generates impulses at a rate of less than 60 beats per minute

II. The rhythm is regular

III. P: QRS ratio is > 1.

IV. They may occur as bigeminy, trigemine or quadrigemine.

V. Vagal stimulation is seen in conditions such as hypothermia, hypovolemia, myocardial infarction.

VI. Treatment is directed at the underlying cause

**7. Which of the above statements regarding sinus bradycardia are correct?**

1. I, II, V, VI
2. I, III, IV, V
3. II, III, IV, V
4. III, IV, V, VI
5. II, III, V, VI


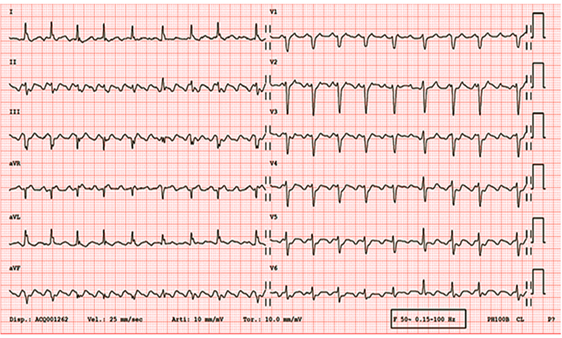


**8. Which of the following arrhythmia is seen in the electrocardiogram above?**

1. Atrial Flutter
2. Atrial Fibrillation
3. Ventricular Tachycardia
4. Ventricular Fibrillation
5. 3rd degree AV block

**9.What is the rhythm in the ECG strip below?**
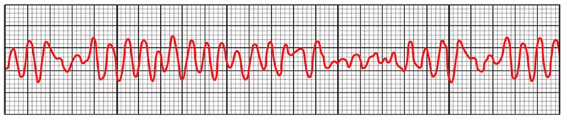


1. Ventricular fibrillation
2. Ventricular tachycardia
3. Idioventricular rhythm
4. Ventricular flutter
5. Accelerated idioventricular rhythm

**10.What is the rhythm in the ECG strip below?**


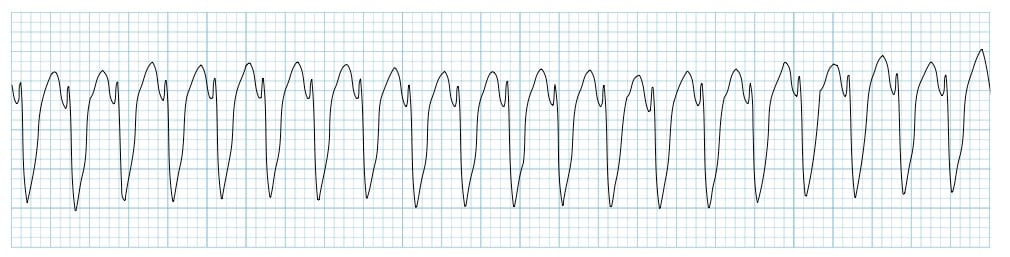


1. Ventricular fibrillation
2. Polymorphic ventricular tachycardia
3. Monomorphic ventricular tachycardia
4. Idioventricular rhythm
5. Accelerated idioventricular rhythm

**11. Which of the following is a shockable (defibrillatory) rhythm?**

1. Pulseless electrical activity
2. Ventricular fibrillation
3. Asystole
4. Normal sinus rhythm
5. Sinus tachycardia

**12.What is the rhythm in the ECG strip below?**


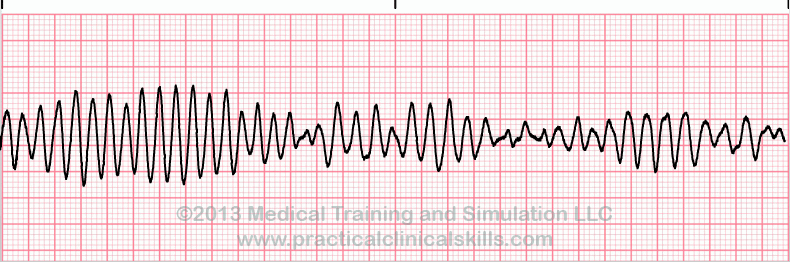


1. Ventricular fibrillation
2. Idioventricular rhythm
3. Monomorphic ventricular tachycardia
4. Torsades de Pointes
5. Accelerated idioventricular rhythm
6. **What is the type of block in the ECG strip below?**?


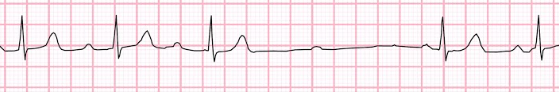


1. 1st Degree AV Block
2. 2nd Degree AV Block Type 1 (Wenckebach)
3. 2nd Degree AV Block Type 2 (Mobitz type 2)
4. 3rd Degree AV Complete Block
5. None

**14.What is the duration of the normal PR interval?**

1. 0.8-0.10 sec
2. 0.20-0.30 sec
3. 0.12-0.20 sec.
4. 0.08-0.10 sec.
5. 0.5-0.8 sec.

**15.What type of block is in the ECG strip below?**


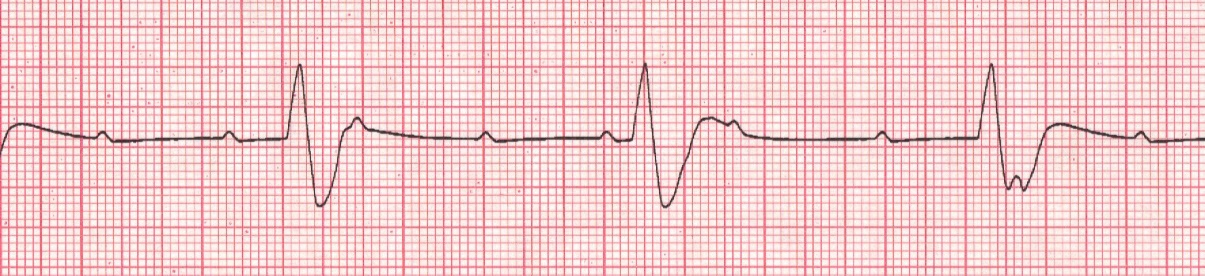


1. 1st Degree AV Block
2. 2nd Degree AV Block Type 1 (Wenckebach)
3. 2nd Degree AV Block Type 2 (Mobitz type 2)
4. 3rd Degree AV Complete Block
5. None
6. **Which of the following is a definite indication for a permanent pacemaker?**
7. Sinus tachycardia
8. Supraventricular tachycardia
9. Non-sustained ventricular tachycardia
10. 1st Degree AV Block
11. 3rd Degree AV Complete Block
12. **What deflections are indicated by the red arrows in the ECG strip below?**


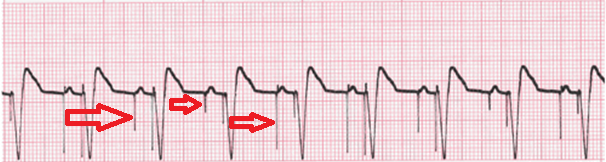


1. P Wave
2. Pacemaker spikes
3. PR Interval
4. QT interval
5. Calibration strips
6. **What is the aim of treatment in the Emergency Department in Acute Coronary Syndrome?**
7. Controlling pain and comforting the patient
8. Trying to keep the infarct area small
9. Preventing death due to arrhythmia
10. Treating complications, if any.
11. All
12. **In which acute coronary syndrome would cardiac enzyme elevation not be expected?**
13. Unstable angina
14. ST elevation Myocardial infarction
15. Myocardial infarction without ST elevation
16. Sudden cardiac death
17. None of them
18. **Acute Coronary Syndrome (ACS)Which of the following information is incorrect?**
19. In patients suspected of having ACS, a 12-lead ECG should be taken and interpreted within 10 minutes of presentation to the emergency department.
20. ST depression, transient ST elevation, and new negative T waves may occur in patients with non-ST elevation ACS.
21. It is known that women diagnosed with ACS report symptoms of indigestion, palpitations, nausea, numbness in the hands, and atypical fatigue more frequently than chest pain, without attributing it to a cardiac cause.
22. Myoglobin;. It is the “earliest rising enzyme” after infarction. It has high cardiac specificity.
23. Patients with ST elevation detected on ECG are candidates for primary coronary angioplasty or thrombolytic therapy.
